# Annual Primary Care 2030 Convening: Creating an Enabling Ecosystem for Person-Centered Primary Healthcare Models to Achieve Universal Health Coverage in Low- and Middle-Income Countries

**DOI:** 10.5334/aogh.2948

**Published:** 2020-08-20

**Authors:** Jessica L. Alpert, Sofiat Akinola, Edward Booty, Dessislava Dimitrova, Thu T. Do, Audu Lucky Emmanuel, Pascal Fröhlicher, Caroline W. Gitonga, Phan Le Thu Hang, Lindsay Hunt, Salim Hussein, Helen Kiarie, Sejal Mistry, Ruth Ngechu, Irina A. Nikolic, Agatha Olago, Todd M. Pollack, Ruben Vellenga, Bram P. Wispelwey, David B. Duong

**Affiliations:** 1 Center for Primary Care, Harvard Medical School, Boston, MA, US; 2 World Economic Forum, Geneva, CH; 3 Reach52, SG; 4 Novartis Global Health, Novartis, Munich, DE; 5 Federal Tertiary Hospital, Taraba State, NG; 6 Integrated Universal Health Management and Innovation Center, Abuja, NG; 7 Program in Global Primary Care and Social Change, Department of Global Health and Social Medicine, Harvard Medical School, Boston, MA, US; 8 Philips Africa Innovation Hub, Nairobi, KE; 9 Department of Planning and Finance, Ministry of Health, Hanoi, VN; 10 Department of Primary Health Care, Ministry of Health, Nairobi, KE; 11 Division of Monitoring & Evaluation, Ministry of Health, Nairobi, KE; 12 ACCESS Health International, SG; 13 Living Goods, Nairobi, KE; 14 The World Bank Group, Washington, DC, US; 15 Division of Primary Health Services and Family Medicine, Ministry of Health, Nairobi, KE; 16 The Partnership for Health Advancement in Vietnam, Beth Israel Deaconess Medical Center, Hanoi, VN; 17 Department of Medicine, Beth Israel Deaconess Medical Center, Boston, MA, US; 18 United Nations Resident Coordinator’s Office, Nairobi, KE; 19 United Nations Population Fund, Nairobi, KE; 20 Division of Global Health Equity, Brigham & Women’s Hospital, Boston, MA, US

## Abstract

**Background:**

The 2019 United Nations General Assembly High-Level Meeting on Universal Health Coverage and the 2018 Declaration of Astana reaffirm the highest level of political commitment by United Nations Member States to achieve access to health services and primary healthcare for all. Both documents emphasize the importance of person-centered care in both healthcare services and systems design. However, there is limited consensus on how to build a strong primary healthcare system to achieve these goals.

**Methods:**

We convened a diverse group of global stakeholders for a high-level dialogue on how to create a person-centered primary healthcare system, using the country examples of the Republic of Kenya and the Socialist Republic of Vietnam. We focused our discussion on four themes to enable the creation of person-centered primary healthcare systems in Kenya and Vietnam: (1) strengthened community, person and patient engagement in subnational and national decision making; (2) improved service delivery; (3) impactful use of innovation and technology; and (4) meaningful and timely use of measurement and data.

**Findings:**

Here, we present a summary of our convening’s proceedings, with specific insights on how to enable a person-centered primary healthcare system within each of these four domains.

**Conclusions:**

Following the 2019 United Nations General Assembly High-Level Meeting on Universal Health Coverage and the 2018 Declaration of Astana, there is high-level commitment and global consensus that a person-centered approach is necessary to achieve high-quality primary healthcare and universal health coverage. We offer our recommendations to the global community to catalyze further discourse and inform policy-making and program development on the path to Universal Health Coverage by 2030.

## Introduction

The 2018 ratification of the Declaration of Astana [1], the 2019 World Health Assembly Primary Healthcare (PHC) resolutions and the 2019 United Nations (UN) General Assembly High-Level Meeting (UN HLM) on Universal Health Coverage (UHC) [2] brought PHC to the forefront of policy conversations and catalyzed a renewed interest in advancing primary healthcare for all. The lack of consensus on how to operationalize the vision of health for all prompted the Harvard Medical School Program in Global Primary Care and Social Change to host a 2018 convening centered on developing an ecosystem for disruptive primary care innovation [3]. The high-level meeting concluded that PHC is the basis for quality health service delivery and has a critical role in achieving UHC.

In 2019, the Harvard Medical School Program in Global Primary Care and Social Change, in partnership with the World Economic Forum and the World Bank convened the second annual high-level dialogue entitled *Primary Care 2030* to further explore the connection between PHC and UHC and the strategies for operationalizing the two synergistically, using the Republic of Kenya and the Socialist Republic of Vietnam as examples. These countries were selected given that the conveners of the meeting have excellent working relationships and existing projects in both countries. In order to foster an informed and contextually relevant discussion, the workshop convened delegations from Kenya and Vietnam that consisted of representatives from each country’s ministry of health, as well as representatives from the UN, non-governmental organizations (NGOs), the private sector, and academia who are active in those countries. In addition to these delegations, a diverse group of expert stakeholders was invited to represent various international organizations, private-sector organizations, and advocacy groups (i.e., patient representation groups, community-based organizations, etc.).

Given the broad scope of the Declaration of Astana and the UN HLM on UHC, the goal for this second meeting was to center discussions on a more focused area of both documents that all stakeholders can embrace: person-centered care. According to the World Health Organization (WHO), person-centeredness involves a recognition of the importance of people, processes, systems, power relations, and values in the foundation and pillars of any effort to improve health and wellbeing. Furthermore, integrated people-centered health services means putting people and communities, not diseases, at the center of health systems, and empowering people to take charge of their own health rather than being passive recipients of services [4]. Although the WHO proposed this definition of person-centered care, there is limited application of person-centered care in low- and middle-income countries (LMICs) [5,6]. The governments of Kenya and Vietnam, in particular, are making strides toward achieving UHC, but have limited plans for how to implement person-centered care. Additionally, there exists a variety of definitions and terms relating to person-centered care, including, but not limited to ‘patient-centered,’ ‘people-centered,’ ‘empowerment,’ ‘involvement,’ ‘patient engagement,’ etc. Although these terms share the underlying theme of facilitation and strengthening of the role of those using services as co-producers of health, many have subtly different meanings that are not rigorously defined [7,8].

Through the convening, we identified four priority areas for diverse stakeholders to engage in, which foster an enabling ecosystem for person-centered primary healthcare. Here, we summarize our convening, adding to the global discourse of how to actualize the Declaration of Astana and the 2019 UN HLM on UHC. Full proceedings are accessible at https://ghd-dubai.hms.harvard.edu/files/ghd_dubai/files/primarycare2030-updated.pdf.

## Creating an Enabling Environment for Person-Centered Primary Healthcare

Translating the Declaration of Astana and the UN HLM on UHC into action at the national and subnational levels is a challenge for many countries. To aid in the translation of these high-level documents into actionable items, the WHO is currently developing the Declaration of Astana Operational Framework, comprised of 14 strategic and operational levers with actions and interventions proposed for each lever. The framework is intended to be adaptable, responsive, and flexible to countries’ needs as they work to achieve UHC. At our convening, we reviewed this Operational Framework along with other frameworks, notably from the World Bank and the United States Agency for International Development (USAID), and specifically discussed how to adapt these frameworks in Vietnam and Kenya through a person-centered approach. Building upon these frameworks, our convening identified four domains that have a critical role in advancing person-centered primary healthcare overall, but especially within the Vietnam and Kenya contexts: (1) strengthened community, person and patient engagement in subnational and national decision making; (2) improved service delivery; (3) impactful use of innovation and technology; and (4) meaningful and timely use of measurement and data.

### Strengthened Community, Person, and Patient Engagement in Subnational and National Decision Making

Although healthcare contexts vary widely, our convening believes that the principles of person-centeredness are universal. This is recognized in the Declaration of Astana and the UN HLM on UHC, yet its implementation at the subnational and national levels in LMICs has been limited [5,6]. The Operational Framework calls for a participatory process, empowering people to define their own priorities and solutions which are incorporated at all levels of government. However, the Operational Framework offers few specific examples of such processes. Our convening suggests three levels of person empowerment, with examples for each level. At the first level, people are empowered in their own healthcare and to be their own health advocates. At this level, people are encouraged to recognize their health needs, seek care, and become engaged members of their care teams. In the second level, people can be empowered at the level of clinical improvement. Here, people are encouraged to identify and prioritize ideas for healthcare improvement through the creation of and participation in people and family advisory committees at healthcare facilities. At the third level, people can be empowered as leaders through the creation of health committees and community advisory panels at the subnational and national levels, which would entail reviewing population health data, co-setting health priorities, and developing participatory research programs. We concluded that national and subnational governments are responsible for engaging people, families, and communities; however, people, families, and communities should also be self-empowered to advocate for their own involvement. We recognize that there are several challenges when involving people, families, and communities. For example, the groups easiest to engage with may be those that are strongly motivated by a single issue (i.e., single disease focus), claim of negligence, or bring a very critical perspective that is not shared by the general population. Our convening affirms that it is important for governments to engage with these groups in order to hear their opinions and to reiterate that person-empowerment and engagement are governmental priorities. Another challenge is financing for person, family, and community engagement. This is an area that may be supported by NGO and community-based organizations (CBO) as part of their missions. As a result of our convening, the delegations from the Kenyan and Vietnamese Ministries of Health committed to formalizing the process of person, family, and community engagement. Kenya will start with a period of public comments online prior to formalizing its National Primary Healthcare Strategic Framework, and Vietnam will include CBOs in stakeholder forums and policy consultations at the subnational and national levels.

### Improved Service Delivery

Our convening recognized the availability of high-quality services at the PHC level as a key component to improving PHC. Despite the inextricable link between the quality of care and PHC, attention and funding have historically prioritized access to care alone [9]. Yet, of the approximately 8.6 million avoidable deaths per year in 137 LMICs, 3.6 million are people who did not access the health system, whereas 5.0 million are people who sought and had access to services but received poor-quality care [10]. Poor-quality care not only leads to adverse outcomes in terms of high morbidity and mortality but also impacts patient experience and patient confidence in health systems [11]. Less than one-quarter of people in LMICs believe that their health systems work well [11]. In Kenya and Vietnam, chronic underfunding of PHC staff and services and prioritization of investments for hospital services have contributed to a lack of public trust and negative perception of PHC. This results in patients bypassing the PHC level and overcrowding secondary and tertiary level hospitals. We recognized that two key actions are needed to improve service delivery at the PHC level: 1) increasing the number of health staff and their capabilities and 2) improving service accessibility and availability. First, to strengthen staff capacity, we suggest that countries implement new training protocols that include an easily accessible and frequently updated competency-based curriculum as well as job aides to support frontline health workers. Second, given that service accessibility and availability at the PHC level is often limited, our convening underscored the importance of assisting countries in developing a basic health services package (BHSP) for the PHC level, coupled with an investment in equipment, diagnostics, and medications to fulfill the BHSP. For example, in Vietnam, less than 25% of PHC facilities offer preventive services for non-communicable diseases (NCDs), and there is limited availability of NCD medications [12]. In Kenya, less than 20% of essential NCD medicines are available at public and private healthcare facilities [13]. Further, while standard treatment guidelines do not restrict the management of common NCDs to specific levels of care, the national essential medicines list restricts availability to sub-district hospitals and higher-level facilities. We recognize the need to involve the private sector and NGOs in a coordinated approach to increasing frontline staff capabilities and improving service accessibility and availability; however, we emphasize that it is the government’s responsibility to endorse training curricula and set regulations, standards and the benchmark around quality healthcare services and delivery.

### Impactful Use of Innovation and Technology

Disruptive innovation and digital technology, which allow for more radical, leapfrog solutions that accelerate development by skipping often inferior, more costly, and less efficient approaches, are required to realize the Declaration of Astana and the UN HLM on UHC [14]. In order to ensure that innovation and digital technology effectively advance person-centered UHC, it is important that digital health solutions align with country needs, because oftentimes, there is a disconnect between the developed technologies and implemented innovations, and the actual needs of the country and their communities. Currently launching in East Africa, the World Bank Group’s TechEmerge ‘match-making’ program has connected 37 companies to 30 diverse healthcare providers through over 40 projects in India and Brazil, helping fill government needs for meeting UHC. However, innovation should also be thought of in a broader sense as realizing new ways of doing business, including changing the processes in which healthcare is delivered and administered. We agreed that implementers should first consider healthcare goals. Then, they should assess how new technologies or processes can help achieve that vision, with consideration for how end-users, patients, and families can have a role in co-creating, co-implementing, and co-assessing the innovations that have been adopted. Our convening also emphasizes that new solutions must take into consideration existing technologies. For example, in Kenya, a community health worker was using two phones for two apps that were both collecting the same information and performing the same analysis, but for different projects. This complicates the work for the frontline health worker, raises questions about double reporting, and detracts from healthcare delivery. Our convening agreed on four policy priorities for incorporating the human-centered approach to innovations in PHC. First, interoperability and use of open-source technology are needed because technology should be utilized to the extent possible for multiple healthcare applications. Second, before proposing solutions, it is necessary to obtain the end-user perspective through methodologies such as the patient pathway analysis (PPA) to identify “pain points,” which can be alleviated [15]. Third, there needs to be a commitment to and framework for information and data sharing among stakeholders that articulate data ownership responsibilities between governments, patients, and technology partners. Finally, a standardized process for innovation assessment and scaling is needed in order to advance innovations beyond the pilot phase. This fourth priority will require close coordination with government partners. Although it is tempting to rely on the promise of innovation and technology to realize health for all, our convening cautions against the excessive focus on health technologies, digitalization, and evidence-based interventions at the expense of person-centeredness.

### Meaningful and Timely Use of Measurement and Data

Our convening recognized that health data in LMICs is often cumbersome, time-consuming, and inefficient to collect, and often not used for real-time decision making. Our group identified three actions that promote the meaningful and timely use of health data to strengthen PHC. First, systems for data generation must be strengthened and innovated upon to ensure accessibility and timely utilization. Programs such as the Primary Health Care Performance Initiative (https://improvingphc.org/) have compiled existing data into a ‘PHC Vital Signs Profile’ dashboard, which allows for better visualization, tracking, and intra-and inter-country comparisons. The Kenya Health and Research Observatory is another example in which government and academia collaborate to provide a ‘one-stop-shop’ that organizes existing health data into accessible visuals that incorporate analytics and promote ‘real-time’ utilization. In Vietnam, the Health Strategy and Policy Institute is the central government organization for collecting and analyzing data to inform policy making. This data is made accessible to government bodies, academic and non-governmental organizations. However, in all three cases, the initiatives are limited by their reliance on data that is often dated, time-consuming, and resource-intensive to obtain. Second, we recognize that existing health indicators do not adequately and effectively measure person-centeredness. Current measurements exist in the form of service utilization data and patient satisfaction scores; however, we believe that measures should be more nuanced and based on patient outcomes that reflect patient goals, ability to function, and meaningful contribution to society. To achieve this, patients and families should be involved in the design and review of these health indicators so that they have a role in assessing progress in their communities as well as in ensuring that decisions based on data and measurement are enacted in a timely manner. Third, our convening emphasizes the role of the private sector, meaningfully engaging in both the collection and reporting of health data. Although over 50% of PHC is delivered by private facilities (i.e., clinics, pharmacies) in Kenya and Vietnam, these private facilities do not all report into the national data system [16,17,18]. Additionally, the health indicators implemented in the private sector supported projects often do not align with those used by government entities. Our convening recommends that the role of government is to establish a set of key PHC indicators and a framework for data collection and sharing through a consultative process with stakeholders. This will serve as a common framework and process by which public, private and non-governmental organizations collect and share PHC health data.

## Limitations

While we believe that the proposed recommendations would help to foster an enabling, person-centered ecosystem for PHC, there are potential barriers that could limit their implementation in LMICs. For example, countries may be limited in digitization and other innovation due to a lack of reliable internet access, and countries may experience difficulty in engaging a diverse representation of community members who have competing interests, lack of organization or representation of community members, and financing for the participation of community members.

For this convening, we brought together a diverse group of global stakeholders for a high-level dialogue on how to create an enabling ecosystem for person-centered primary healthcare. However, this was by no means representative of all stakeholders, as we focused on only two countries, nor was it representative of all relevant topics (e.g., primary healthcare financing). Additionally, our patient representative did not represent any particular patient group, and her experience is shaped by the US and Swedish healthcare systems. The “private sector” in our convening predominately represents multinational corporations, rather than private-sector service delivery providers, which exist in many LMICs. Given the limited scope of the private sector representation in our convening, we did not discuss the “private sector-ization” of healthcare services; rather, we focused on how public-private partnerships can meaningfully contribute to the national or subnational PHC agenda, aligning objectives, strategies and shared indicators for success. The summary from our convening is not intended to be representative of all global voices, but rather to offer a concise perspective from some engaged voices.

## Conclusions

While we celebrate the renewed high-level political declarations and commitments to PHC, UHC, and health for all, we also acknowledge our shortfalls and the challenges ahead in the implementation and actualization of these high-level commitments. We want to capitalize on this chance to advocate for a PHC system that is truly person-centered. Today, shared global threats of new infectious diseases, climate change, political unrest, and economic uncertainty will only exacerbate the growing chasm of health inequities. Building a PHC system that is person-centered will increase the resiliency of health systems to confront these threats, and is critical to achieving UHC and the vision of health for all.
